# In Vitro Methodologies to Study the Role of Advanced Glycation End Products (AGEs) in Neurodegeneration

**DOI:** 10.3390/nu14020363

**Published:** 2022-01-15

**Authors:** Marialena Chrysanthou, Ignacio Miro Estruch, Ivonne M. C. M. Rietjens, Harry J. Wichers, Tamara Hoppenbrouwers

**Affiliations:** 1 Department of Food Chemistry, Wageningen University and Research, Bornse Weilanden 9, 6708 WG Wageningen, The Netherlands; harry.wichers@wur.nl; 2 Department of Toxicology, Wageningen University and Research, Stippeneng 4, 6708 WE Wageningen, The Netherlands; ignacio.miroestruch@wur.nl (I.M.E.); ivonne.rietjens@wur.nl (I.M.C.M.R.); 3 Food and Biobased Research, Wageningen University and Research, Bornse Weilanden 9, 6708 WG Wageningen, The Netherlands; tamara.hoppenbrouwers@wur.nl; 4 Department of Food Quality and Design, Wageningen University and Research, Bornse Weilanden 9, 6708 WG Wageningen, The Netherlands

**Keywords:** advanced glycation end products, AGEs, neuro–immune axis, inflammation, neurodegeneration, neuroinflammation, blood–brain barrier, in vitro models

## Abstract

Advanced glycation end products (AGEs) can be present in food or be endogenously produced in biological systems. Their formation has been associated with chronic neurodegenerative diseases such as Alzheimer’s disease, Parkinson’s disease, multiple sclerosis, and amyotrophic lateral sclerosis. The implication of AGEs in neurodegeneration is related to their ability to bind to AGE-specific receptors and the ability of their precursors to induce the so-called “dicarbonyl stress”, resulting in cross-linking and protein damage. However, the mode of action underlying their role in neurodegeneration remains unclear. While some research has been carried out in observational clinical studies, further in vitro studies may help elucidate these underlying modes of action. This review presents and discusses in vitro methodologies used in research on the potential role of AGEs in neuroinflammation and neurodegeneration. The overview reveals the main concepts linking AGEs to neurodegeneration, the current findings, and the available and advisable in vitro models to study their role. Moreover, the major questions regarding the role of AGEs in neurodegenerative diseases and the challenges and discrepancies in the research field are discussed.

## 1. Introduction

**Figure 1:**
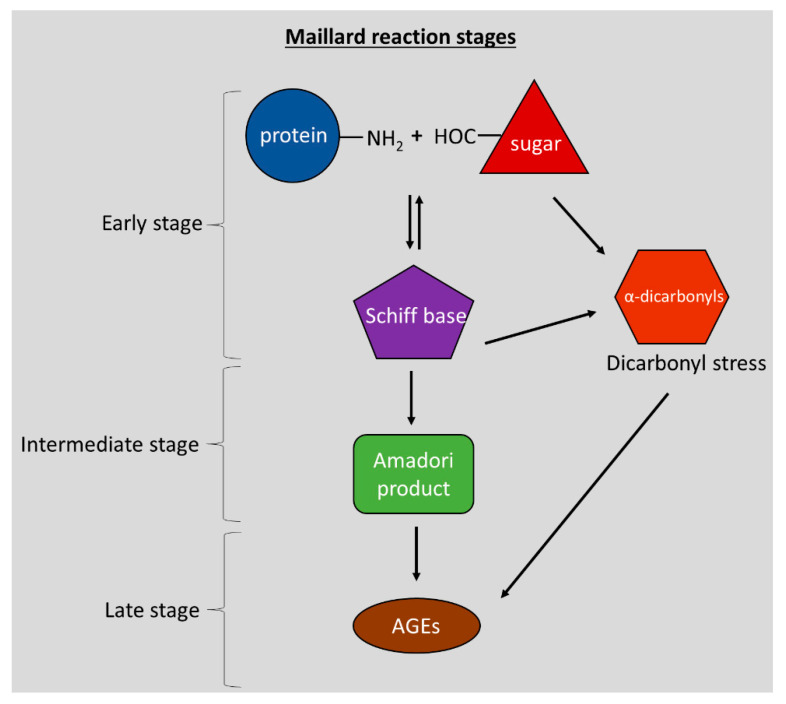
The three stages of Maillard reaction and the formation of AGEs and α-dicarbonyls.

Louis Camille Maillard, a French Biochemist, was the first to observe the reaction between amino acids and carbohydrates, which resulted in the formation of brown-coloured compounds [1]. This reaction would later become known as the Maillard reaction (MR). Several decades later, in 1953, John Hodge published his research on Maillard reaction products (MRPs). He was the first to prove that the reaction could occur in food, and he described the stages of the MR: the early, the intermediate, and the advanced stage [2]. The early-stage consists of a reaction between the amino group of a protein or amino acid with reducing sugar, e.g., glucose or fructose, which results in a so-called Schiff base. From the Schiff base, reactive α-dicarbonyl compounds and oxoaldehydes can be generated such as glyoxal (GO), methylglyoxal (MGO), and 3-deoxyglucosone (3-DG). In the intermediate stage, the more stable Amadori product is formed. Finally, in the advanced stages, further oxidation and molecular rearrangements of the Amadori product result in the formation of protein adducts and cross-links of proteins known as advanced glycation end products (AGEs). Due to their high reactivity, MR-derived α-dicarbonyl compounds react and create cross-links with proteins resulting in protein damage. Hence, the term “dicarbonyl stress” is often used to refer to the damaging effects of α-dicarbonyls on cellular proteins. AGEs comprise a group of complex and heterogeneous compounds such as pyrraline, hydroxymethylfurfural (HMF), Nε-(carboxyethyl)-lysine (CEL), Nε-(carboxymethyl)-lysine (CML), Nδ-(5-hydro-5-methyl-4-imidazolon-2-yl)-ornithine (MG-H1), pentosidine, fructosyl-lysine (FL) and glucosepane (see Figure 1) [3,4,5]. In biomedical research, the acronym AGEs became popular to refer to the MR’s late-stage products because their presence in the human body correlates with aging and neurodegeneration/neuroinflammation [6].

Diet can be an essential source of AGEs (dietary AGEs) [7]. AGEs have been extensively studied in the food industry because the sensory characteristics of processed foods, such as texture, aroma, and flavour, are related to Maillard reaction products (MRPs) [8]. It was only in the mid-1950s when it was shown that the same reactions could also occur in vivo in the human body [9,10]. In 1955, Kunkel and Wallenius documented the presence of modified hemoglobin in diabetic patients [10]. The term “non-enzymatic glycation” or simple “glycation” would be later used to describe the in vivo reaction of amino acids with glucose in the absence of enzymes, distinguishing the glycation from the enzymatic glycosylation [11,12]. Glycation naturally occurs in biological systems at moderate temperatures (37 °C) following the constant co-existence of proteins with sugars (endogenous AGEs). Thus, AGEs can enter the body via ingestion of thermally processed food or be endogenously produced, with kinetics depending on the type of AGE [13]. AGEs accumulate in the human body throughout life, especially in aging tissues [14]. Excessive accumulation of AGEs can negatively affect health [15,16]. As mentioned, the consumption of food products with high content of AGEs (or their intermediate precursors) is one of the ways that AGEs enter the human body. In this context, the AGE load of several food products was investigated and AGE food databases were generated [17,18,19]. Despite the direct intake of AGEs via AGE-rich food products, a high glycemic diet also promotes the formation of endogenous AGEs due to increasing levels of glucose which acts as a glycating agent. For example, diabetes is associated with increased levels of AGEs [20]. In this context, hyperglycemic conditions such as diabetes promote the glycation process and the accumulation of AGEs [20]. Moreover, increased glycolysis leads to elevated levels of glycolysis by-products such as MGO, an AGE precursor [21,22]. Interestingly, epidemiological research has suggested the association of type 2 diabetes with an increased risk for neurodegenerative diseases (ND) [23].

The excretion mechanisms of AGEs from the body is an important factor to consider in the assessment of their effect on human health. Once ingested the dietary AGEs will be digested and partially absorbed in the intestine. Then, they will enter the circulation or accumulate in tissues or they will be excreted via urine or feces [24]. Human and animal trials have shown that increased intake of dietary AGEs (i.e., CML, CEL, MGH-1) was associated with increased levels of AGEs in circulation and accumulation of AGEs in tissues [25,26,27]. In addition, human studies have shown that only 10% of dietary AGEs are absorbed in the gastrointestinal tract from which approximately 66% remains in the body and 33% gets eliminated via urine [28,29]. The part of absorbed dietary AGEs will eventually merge into the pool of endogenous AGEs increasing the load of circulating AGEs.

Under homeostatic conditions, the AGE-precursors dicarbonyls are maintained at low levels due to the glyoxalase system. However, during aging the dicarbonyl levels increase, promoting the formation of AGEs [30]. Glyoxalase is a dicarbonyl-detoxifying system composed of two enzymes: Glyoxalase I (GLO1) and Glyoxalase II, (GLO2). The glyoxalase system prevents the accumulation of reactive dicarbonyls and therefore the formation and accumulation of AGEs in tissues [31]. GLO1 and GLO2 convert methylglyoxal/glyoxal to D-lactate/glycolate respectively [32]. GLO1 has been an especially important topic of research in aging and neurodegeneration [33]. In the brain, the mRNA levels, protein expression and activity of GLO1 are decreased with aging [34]. Murine AD transgenic brains showed increased levels of GLO1 as a response to tau aggregation [35]. In addition, GLO1 expression was increased in human early- and middle-stage AD-derived neurons but not in late-stage AD patients [36].

Taking all the above into consideration, it is clear that individuals with age-related chronic diseases (i.e., chronic kidney disease, diabetes) in which excretion mechanisms do not function properly might be at higher risk than healthy individuals.

Although clinical studies have suggested the relationship between AGEs and ND, there is still little scientific understanding of the modes of action (MoAs) by which AGEs promote neurodegeneration. For example, the brain’s immune response to AGEs and the effect of this response on neuronal homeostasis are not fully understood. To obtain insights in MoAs clinical studies or in vivo studies with experimental animals could be considered, but ethical drawbacks and confounding factors may hamper the use of these in vivo approaches in mechanistic studies. All these limitations point to the potential of in vitro models to study the role of AGEs. Hence, this paper aims to present the current findings in research regarding AGEs and neurodegeneration, explain the relevant concepts, and review currently available in vitro cell models that are relevant and could be considered to study the role and the MoAs of AGEs in neurodegeneration. In vitro methodologies pertinent to AGEs and the neuro-immune axis will be presented, from simple traditional cell cultures to more complex three-dimensional (3D) model systems. The review will also overview the advantages and disadvantages of the available in vitro models, the main directions for future research and the challenges in the research field.

## 2. AGEs and Neurodegeneration

### 2.1. Evidence Associating AGEs with Neurodegeneration

The role of AGEs as accelerators of ND was suggested based on clinical data from diseased individuals. Since the identification of the production of AGEs in biological systems, a growing body of literature recognises the implication of AGEs in diseases related to aging, including ND such as Alzheimer’s disease (AD), Parkinson’s disease (PD), amyotrophic lateral sclerosis (ALS), and multiple sclerosis (MS) [37,38,39,40,41,42,43]. Human clinical studies have shown increased levels of AGEs in the serum of AD patients [44]. Immunostainings of AD patients’ brain tissues were found to have three times higher AGE content than the brain tissues of healthy matched controls [38,45]. In addition, increased levels of CML and MG-H1 were found in the cerebrospinal fluid (CSF) of AD and MS patients [42,46,47]. Furthermore, dicarbonyl AGE precursors can cause the cross-linking of α-synuclein while they were found to co-localise with Lewy bodies of PD patients [39,48,49]. In AD, the aggregation of human β-amyloid peptides and the formation of senile plaques are major pathophysiologic characteristics of the disease. Interestingly, it was observed that AGEs enhance the expression of amyloid precursor protein (APP) expression by inducing reactive oxygen species (ROS) and enhancing the cross-linking of β-amyloid protein, which accelerates the amyloid plaque formation due to the increased fibrilisation [50,51]. Hence, a large number of clinical studies have shown the accumulation of AGEs in the brain or the circulation of ND patients, but the mechanisms that underpin these observations are not well understood.

### 2.2. Concepts in AGE-Mediated Neurodegeneration

The blood–brain barrier (BBB) is a physical and functional barrier that separates the systemic circulation from the brain. The BBB’s function is to regulate the influx and efflux of molecules from the periphery to the brain and vice versa, protecting the brain from pathogens and molecules that could initiate inflammatory responses that can cause neurotoxicity and neuronal degeneration [52]. Several cell types are involved in the formation of the BBB. The primary cell type in BBB is the brain endothelial cells characterised by the high expression of tight junctions that regulate the barrier’s integrity and the passive paracellular diffusion of molecules [53]. The brain endothelium is surrounded by pericytes, glia, and neurons that contribute to the BBB [54]. BBB disruption is a common feature in ND [55]. As mentioned before, in ND, the increased presence of AGEs in the brain and systemic blood circulation was found. An animal study with mice fed with isotope labelled CML showed that CML could cross the BBB and accumulate in murine brains [26]. Ex vivo, in vivo, and in vitro experiments have shown that AGEs can disrupt the BBB by causing a decrease in the expression of tight junction proteins (claudin-5 and occludin), increased permeability, and mitochondrial metabolism impairment of brain endothelial cells [56,57,58,59].

A hallmark of ND is neuroinflammation in which microglia play a significant role. Microglia are the brain’s resident macrophages, and they constitute 0.5–16.6% of the total brain cell population, depending on the area of the brain [60]. They form the first-line immune defense of the brain, and their role is to survey the brain’s microenvironment, detect potential infectious molecules and protect the central nervous system [61]. In addition, microglia have an essential role in regulating neuronal synapsis and excitability by releasing cytokines, reactive oxygen species (ROS), nitric oxide (●NO), and neurotrophic factors [62,63]. Under the absence of stimuli, microglia are in the resting state, also known as the M0 state. However, the resting state is rather a dynamic state than a dormant one. Due to their high motility, even in the resting M0 state, microglia can sense changes in the brain microenvironment [64]. Inhibitory signals from neurons keep the microglia in the M0. Once exposed to an inflammatory stimulus, such as bacterial lipopolysaccharide (LPS) or protein aggregates, microglia get activated, followed by morphological changes; from the resting ramified morphology, they transform into the activated amoeboid form [65]. Depending on the stimulus, a variety of pro-inflammatory molecules such as interleukin-1β (IL-1β), interleukin-6 (IL-6), and tumor necrosis factor-α (TNF-α) are secreted by activated microglia [66]. Two activated microglial phenotypes have been described, the pro-inflammatory M1 and the anti-inflammatory M2 phenotype. In vitro experiments on microglia exposed to pro-inflammatory mediators such as interferon (IFN-γ) and LPS have shown that upon these exposures, the cytokine profile is driven towards the M1 phenotype. In contrast, the incubation of microglia with anti-inflammatory cytokines such as interleukin-4 (IL-4) drives them towards the M2 phenotype [67].

Although the pathophysiology among the distinct NDs is different, neuroinflammation is a common characteristic shared by all NDs [68,69]. However, whether neuroinflammation is the cause or the consequence of neurodegeneration remains still to be elucidated. Although microglia’s primary role is to protect the brain, persistent activation of microglia accompanied by secretion of pro-inflammatory mediators and increased oxidative stress by ROS production initiate a cascade of pro-inflammatory signalling events that can have detrimental effects on brain homeostasis. Impaired and chronic microglial activation plays a crucial role in neuroinflammation and has been associated with various NDs [70,71,72,73,74]. Activated microglia were found to surround Lewy bodies and amyloid plaques in PD and AD patients [75,76,77]. Increased activated microglia levels were observed in ALS patients’ spinal cord [78,79,80]. Moreover, activated microglia and Aβ accumulation were found to co-localise in AD brains [81,82].

AGEs are amongst the candidate molecules that accumulate in the brain tissues of patients with NDs and can cause a pro-inflammatory response by microglia. AGE-rich senile plaques in the AD brain are surrounded by activated microglia, which secrete a variety of inflammatory compounds which can also compromise and disrupt the BBB [75,76,83,84,85].

From a mechanistic perspective, AGE-related disruption of cell homeostasis may proceed in two ways: (1) binding of AGEs to receptors for AGEs, which leads to a cascade of cellular events, and (2) formation of reactive AGE precursors causing the dicarbonyl stress and creating cross-links with intracellular or extracellular proteins and therefore impair protein functionality [86]. The interaction of AGEs with the receptor for AGEs (RAGE) plays a key role in activating microglia [87]. Interaction of AGEs with RAGE causes activation of a signalling cascade, which leads to increased ROS formation via the activation of NADPH oxidase (NOX), an essential enzyme in the regulation of inflammation [88]. Following the NOX activation, the activation of the transcriptional factor nuclear factor kappa B (NF-κΒ) occurs, which is involved in the expression of pro-inflammatory cytokines [89,90,91]. Recent studies have shown that AGEs can drive macrophages and microglia towards the pro-inflammatory M1 phenotype while inhibiting the AGE-RAGE signalling pathway by a RAGE blocker (FPS-ZM1) or a Rho/Rho kinase (ROCK) inhibitor (fasudil) led to an M2 phenotype [87,92]. Concerning the BBB disruption by AGEs, in vivo animal studies have shown that RAGE signalling is involved in BBB damage [93]. Equally important is that some studies have examined the intracellular production of AGEs by microglia [94,95,96,97]. Activated microglia can produce and secrete glycated proteins such as glycated albumin with toxic properties towards primary neurons [97,98,99]. The intracellular formation of AGESs was also observed in neuronal cells [100]. However, in both cases, the detection of AGEs was performed with immunohistochemical assays.

**Figure 2:**
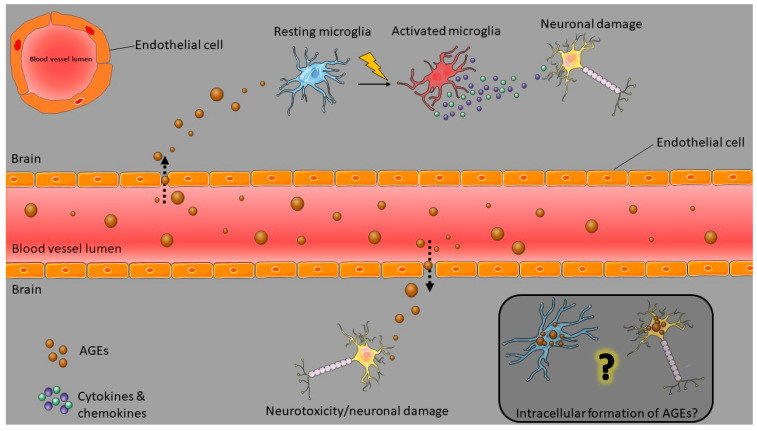
Concepts in AGE-mediated neurodegeneration. Circulating AGEs can disrupt the brain endothelial barrier and enter the brain. There they activate microglia and cause them to initiate an inflammatory response that can affect neuronal survival. AGEs can also have direct cytotoxic effects on neurons. Moreover, some studies have shown the intracellular formation of AGEs in microglia and neurons. However, the mechanisms remain still unclear. This figure was created with Servier Medical Art templates, licensed under a Creative Commons Attribution 3.0 Unported License; Available online: https://smart.servier.com (accessed on 23 July 2021).

Overall, Figure 2 depicts the two possible ways by which AGEs can affect brain homeostasis and cause neuroinflammation and neurotoxicity. In the first case (upper part of the figure), circulating AGEs can compromise the BBB and cross it, reaching the underlying microglia. Microglia then get activated and release inflammatory substances, which can damage neurons. Alternatively (lower part of the figure), crossing AGEs can reach neurons and exert their cytotoxic effects. To the best of our knowledge, only a few studies have investigated the intracellular formation of AGEs by microglia and neurons. Further studies are required to identify the type of AGEs generated and their role in neuronal damage.

## 3. In Vitro Models to Study the Effect of AGEs on the Neuro-Immune Axis

The pathogenesis of ND is complicated, and many cell types can be involved in the onset and progression of the disease. To date, different in vitro methodological approaches have been applied in studies on the mechanisms behind neuroinflammation and, ultimately, neurodegeneration. As regards AGEs, several researchers have used the available in vitro tools to answer questions related to the implication of AGEs in neurodegenerative diseases. The following sections will describe the most relevant methodologies available in the literature about AGE-mediated induction of neuroinflammation and neurotoxicity, starting from simple approaches including monocultures and building up to more complex co-culture and 3D cell culture models.

### 3.1. Monocultures

#### 3.1.1. Models of Brain Inflammation

##### Microglial Cell Models

Several microglial cell lines are used in research to study neuroinflammation, which can be categorised into immortalised microglia cell lines (retrovirus transformed or non-retrovirus transformed) [101] and primary microglia cultures. The most common animal and human microglial lines are described below.

- Immortalised microglial cell lines

To overcome the burden of laborious cultures of primary cells with a short lifetime, animal and human immortalised microglial cell lines were developed by transforming primary microglial cells.

- Animal-derived microglia cell lines

The microglial cell lines BV2, N9, and N11 are derived from murine brain. The BV2 rat microglial cell line was developed by Blasi et al. by transfecting murine microglia with a raf/v-myc carrying retrovirus (J2) [102]. BV2 cells were assessed for the expression of microglial cell markers, and they were found to be positive for the specific cell membrane antigens MAC-1 and MAC-2. Additionally, they were tested negative for glial fibrillary acidic protein (GFAP) and glucocerebrosides which are markers for astrocytes and oligodendrocytes, respectively. Furthermore, BV2 cells express RAGE [89,103] while they present a phagocytic activity in response to Aβ fibril exposure, and express IL-1 upon LPS stimulation [102,104,105]. In comparative studies between primary rat microglia and the BV2 line, the researchers investigated the ability of BV2 cells to produce activation markers and to release pro-inflammatory compounds while they also characterised their gene expression profile upon activation with LPS [106,107]. The results showed that, like primary cells, the BV2 cell line expressed the microglial activation marker IBA-1, but the release of ●NO upon LPS stimulation was lower in BV2 than the primary cells [106,107]. It was also demonstrated that these cells respond to Aβ fibrils with increased phagocytosis in a dose-dependent manner [104]. Furthermore, the induction of genes upon the stimulation with LPS appeared 90% similar to what was observed for primary cells, but the induction levels were lower in BV2 cells compared to primary microglia [107]. Despite their similarity to primary microglia, a study has shown that these cells present increased proliferation and adhesion [106]. However, due to their similarity with primary cells concerning cytokine secretion profile and accessibility, BV2 is an extensively used cell line model in neuroinflammation and AGEs [89,90,108,109,110,111]. For example, the study by Subedi et al.,showed the induction of oxidative stress by MGO-modified bovine serum albumin (BSA) in BV2 cells, which decreased after the administration of sulforaphane (SFN) [89]. The cells responded to MGO-modified BSA (MGO-BSA) with increased levels of ●NO (in a dose-dependent manner) and ROS, and with increased protein expression of inducible nitric oxide synthetase (iNOS), cycloxygenase 2 (COX-2), NLRP3 inflammasome, nuclear NF-κB, TNF-α, IL-6, mitogen-activated protein kinases (MAPKs), and RAGE [89]. However, under the presence of SFN, the expression of neuroinflammatory mediators and RAGE decreased.

The N9 and N11 mouse microglial cell lines were developed by transforming primary mouse embryonic microglial cells with v-myc or v-mil oncogenes of the avian retrovirus MH2 [112]. The clones were found to be positive for microglial cell surface markers such as FcR, MAC-1, and F4/80, while they appeared to be negative for glial and oligodendroglial markers: GFAP, A2B5, and Gal-C. Additionally, the clones of microglial cells produced IL-6, TNF-α, and IL-1 in response to LPS stimulation. Both lines were shown to express the RAGE receptor [113,114,115]. The N11 line was studied for its ability to express a complete set of pro-inflammatory cytokines upon stimulation [116,117]. Furthermore, this line presented similar phagocytic capacity as primary cells [115]. Moreover, this cell line was used as a model of AGE-induced neuroinflammation. Glycated BSA was shown to induce the activation of the N11 microglial cells [99]. Chicken egg albumin (CEA), glycated with glucose, induced the production of ●NO in a dose-dependent manner and similarly to the induction caused by LPS [114,118]. Moreover, glycated CEA induced the expression of iNOS, TNF-α, and IL-6 [114,118]. In another study, glucose-modified BSA caused an increased NO production in a dose-dependent manner in N11 cells [119]. Another study showed that exposure of N11 cells to glycated BSA increased the secretion of IL-6, monocyte chemoattractant protein (MCP-1), and TNF-a [120].

- Human derived microglial cell lines

Over the last decades, many other immortalised microglial cell lines have been developed from different species [66,101]. Nagai et al. developed the immortalised human cell line HMO6 by transfecting human embryonic primary cells with a v-myc carrying PASK 1.2 retroviral vector [121]. The cytokine response of these HMO6 cells to LPS or Aβ was compared with the response of primary cells [121]. It was found that the exposure of HMO6 cells to LPS induced the expression of inflammatory mediators similarly to the primary cells except for the expression of Macrophage Inflammatory Protein (MIP)-1α and TNF-α, which did not appear to be increased. In addition, the secretion of cytokines in response to LPS or Aβ significantly increased for interleukin-8 (IL-8) and TNF-α, but not for IL-1β, IL-6, and MIP-1α. In the field of glycation, it was demonstrated that HMO6 cells express RAGE [122] and secrete glycated albumin upon exposure to α-synuclein in a PD disease model [96,122]. The HMO6 microglial cell line was also used in another study in which the researchers found that activation of HMO6 cells with Aβ peptides or LPS led to increased albumin production by the microglial cells [96]. Later on, they showed that in Aβ-activated HMO6 cells, albumin co-localised with AGEs (as seen with immunohistochemistry) and therefore concluded that activated HMO6 cells secreted AGE-modified albumin [98]. Furthermore, the expression of RAGE in HMO6 cells increased in a dose-dependent manner following the exposure to Aβ. Nevertheless, the HMO6 cell line has not been extensively used since it was patented by the research group that first developed it.

Another human immortalised microglial cell line is the CHME-5 cell line which was immortalised by transfecting the SV40 virus to primary cultures [123]. Over the years, different names have been used for this cell line, i.e., CHME3 or CHME-3 cells or C13NJ cells or HMC3 cells [124,125,126]. This line was found positive for the myeloid markers CD68 and CD11b and negative for the astrocyte marker GFAP. Furthermore, HMC3 cells tested positive for the microglia/macrophage marker IBA1 and the endotoxin receptor CD14. Incubation of HMC3 microglia with IFN-γ caused an upregulation of microglial activation markers such as MHCII, CD68, and CD11b [127]. In addition, the two polarised microglial phenotypes of M1 (CD40 and CD86 positive) and M2 (CD163 and CD206 positive) were observed in this cell line like in primary microglial cultures [128,129].

Increased expression of IL-6 was observed upon exposure of these cells to LPS or Aβ [130]. The phagocytic capacity of the cells was confirmed when the cells were exposed to Aβ [131]. Moreover, these cells express RAGE [132]. Nowadays, the cell line is commercially available under the name HMC3. In AGE research, the CHME-5 cell line has been used as a microglial cell model. One study tested the effect of the degree of protein glycation on cell cytotoxicity and apoptosis in CHME-5 cells [133]. The researchers showed that all groups of glycated human serum albumin (HSA), from the low to highly modified, were able to induce oxidative stress (measured by •NO production) but only highly modified HSA could affect the cell viability, metabolic activity, and apoptosis of the cells. In addition to that, they showed that incubation of the cells with anti-RAGE antibody prevented the death of cells but did not affect the ROS formation induced by the highly modified AGEs. Another study with CHME-5 cells showed that glycated BSA activated the CHME-5 cells as reflected by increased Glut-5 and CR3/43, markers of microglial activation. The expression of RAGE and the production of TNF-α levels increased in cells treated with glycated BSA, whereas TNF-α was not detectable after preincubation of the cells with anti-RAGE antibody [132].

- Human and animal-derived primary microglial cells

The availability of human primary microglial cells is limited because most human primary cells are derived from embryos or post mortem brain tissue. Therefore, due to ethical and legal issues, obtaining access to these materials is difficult. Animal primary microglial cells are more accessible and, based on studies aimed at characterising primary microglia, it was found that the advantage of primary microglia is the ability of these cells to secrete products and cell surface markers that resemble the in vivo situation. Mammalian primary microglia cultures are found to be negative for GFAP, GC, and peroxidase activity (characteristic of neutrophils) and positive for non-specific esterases, which is characteristic of the microglial population [134]. Furthermore, these microglia have two functionally distinct activation states, the M1 and M2, similar to systemic macrophages [135,136,137].

Primary rat microglial cultures were found to release •NO and superoxide (O_2_•−) via the upregulation of iNOS and NOX, respectively [138]. Furthermore, rat primary microglia express and secrete pro- and anti-inflammatory factors upon exposure to stimuli, similar to what was observed for monocytes, macrophages, and neutrophils in vitro [139]. In AD pathophysiology, the accumulation of Aβ oligomers is a characteristic of the disease. Primary microglial cultures were found able to respond and react to the accumulation of Aβ by initiating their phagocytic activity (M2 phenotype) [140] or the release of pro-inflammatory cytokines such as IL-β, TNF-α, IFN-γ (Μ1 phenotype) [141]. Additionally, increased levels of •NO were produced by microglia as a response to Aβ oligomer accumulation [142]. In a particular study with rat primary microglia, it was shown that the primary cells express RAGE. More precisely, the researchers in this study showed that curcumin inhibited the Aβ induced neuroinflammation by suppressing the expression of RAGE and TLR4 [143]. Another study with primary rat microglia demonstrated that AGE-BSA reduced the viability of microglial cells in a dose-dependent manner and induced the production of intracellular ROS, malonaldehyde (MDA), and NADPH oxidase (NOX2) [144]. In addition, AGE-BSA attenuated the superoxide dismutase (SOD) and glutathione (GSH) and induced the expression of the antioxidant system, as demonstrated by the increased protein expression of Nrf2 and heme oxygenase-1 (HO-1). In addition, it was observed the nuclear translocation of NF-κB and subsequent elevated production of TNF-α, IL-1β, COX-2, and iNOS. The RAGE inhibitor FPS-ZM1, alleviated all the inflammatory effects induced by AGE-BSA and further activated the antioxidant system of Nrf-2 and HO-1 [144,145].

Overall, primary cell cultures facilitate the ability to work with an in vitro model representative for the in vivo situation to study the role of AGEs on the brain’s immune responses. As discussed above, primary microglia produce cytokine markers, and they can also polarise into the two distinct phenotypes observed in vivo. In addition, primary cells retain the genetic integrity and recapitulate the pathophysiology of the disease. Despite their high relevance to the human in vivo state and the fact that primary human cells bypass ethical issues over the use of animals in biomedical research, their growth potential is limited. The extensive, laborious, and costly preparations of these cells, as well as the difficulty in obtaining material due to ethical restrictions, and the short lifetime under in vitro conditions, make them more challenging to work with as compared to other microglial cell models with comparable similar properties, such as immortalised microglial cell lines. The same challenges are met in all kinds of primary cells that are described in the following sections of this review, including primary monocytes, neurons, endothelial cells.

#### 3.1.2. Other Inflammation Models

- Monocytes Cell Models

Although microglia are the most relevant in vitro model to study the brain’s immune responses to AGEs, monocytes have also been used as a model of inflammation to study the effect of AGEs on tissue macrophages. This model might be less relevant to neurodegeneration, but findings from such studies could still provide insights into the possible role of AGEs in neuroinflammation.

##### Immortalised Monocyte Cell Lines

- Human derived monocytic lines

Human blood-derived peripheral monocytes are extensively used in research to generate differentiated macrophages. However, these primary monocytes present individual-dependent variability and low expansion rates [146]. Established in 1980, the THP-1 is a human leukemia monocytic cell line used in research as a model of inflammation, as this cell line can be used as a monocyte model and as a macrophage model [147,148]. In addition, the ability to obtain experimental data with the same genetic background minimises the variability in experiments [148,149,150]. In a comparative study between human primary monocyte-derived macrophages and several myeloid cell lines, the THP-1 line was found to have the most similarities to primary macrophages [149]. Over the past years, a significant number of studies investigated the effect of AGEs on THP-1 cells [92,151,152,153,154,155,156,157,158,159,160]. A study showed that incubation of THP-1 cells with glucose-modified BSA (modified for a period of 6 or 8 weeks) significantly increased the release of vascular endothelial growth factor (VEGF) as compared to cells exposed to non-glycated BSA [151]. THP-1 cells showed a statistically significant increase in VEGF only when they were exposed to extensively glucose-modified HSA (modified for nine weeks) in the same study. However, no release of TNF-α was observed upon exposure to modified BSA [151]. In addition, incubation of THP-1 cells with S100b (a RAGE ligand) induced the gene expression of RAGE, MCP-1, interferon γ inducible protein-10 (IP-10), COX-2 and, NOX2. In addition, the intracellular O_2_•− levels increased [154]. Blockage of the RAGE binding of AGEs with the LR-90 inhibitor has anti-inflammatory effects on THP-1 cells. LR-90 inhibited the S100b and TNF-α induced activation of NF-κB and promoted the switch of macrophages to their anti-inflammatory phenotype (M2) [154].

In another study, THP-1 exposed to hypoxia caused an increase in the secretion of AGEs in the supernatant as measured with ELISA [156]. Hypoxia induces RAGE expression via the activation of NF-κΒ and hypoxia-inducible factor 1-α (HIF1A) [153]. In addition, a study on intermittent hypoxia showed that THP-1 cells enhanced the MCP-1 mediated adhesion and chemotaxis under hypoxic conditions and switched the phenotype towards M1 via upregulating the expression of RAGE and the activation of the Nf-κB pathway [155]. However, the mechanism by which hypoxia causes the secretion of AGEs by THP-1 cells is not fully understood. Similarly, exposure to glycated BSA drove macrophages towards the M1 pro-inflammatory phenotype as reflected by the expression of pro-inflammatory cytokines such as IL-6 and TNF-α, enzymes related to the M1 phenotype (iNOS), M1 surface markers (CD11c and CD86), and RAGE [92]. Incubation of THP-1 cells with MGO led to increased secretion of AGEs and glycation of cell surface proteins, as detected with immunoblotting. However, the expression of RAGE and production of ROS remained unchanged. In addition, the expression of pro-inflammatory cytokines (IL-1β, IL-8, TNF-α) increased, whereas the phagocytic activity was compromised [157]. Incubation of THP-1 cells with MGO led to increased migration of the cells [158]. However, the exposure to MGO showed an impaired chemotactic migration of THP-1 cells towards chemotactic factors as PIGF-1 and VEGF-1. Monocytes migrate into damaged tissues in response to chemotactic molecules. There, monocytes differentiate into macrophages which will release cytokines and other inflammatory mediators. Therefore, the migration of monocytes is an essential factor in inflammation [158]. Moreover, exposure of THP-1 to MGO-modified BSA increased the gene expression of COX-2 [159]. THP-1 cells exposed to glucose and MGO-modified HSA showed that MGO-modified HSA induced the synthesis and secretion of macrophage-colony stimulating factor (M-CSF) and that MGO modified HSA is a more potent inducer of TNF-α secretion compared to glucose modified HSA [152].

- Human and animal-derived primary monocytes

Human monocyte-derived macrophages stimulated with extensively glucose-modified HSA (9 weeks) released significantly higher VEGF, IL-8, TNF-α, and tissue factor antigen levels. However, minimally glycated HSA (5 weeks) did not cause induction of VEGF [151]. Similarly, CML-modified BSA induced the release of TNF-α and VEGF. Interestingly, this study showed that glycation’s efficiency depends on the buffer type in which albumin is dissolved. More precisely, the study proved that glycation in monophosphate buffer (NaH_2_PO_4_) is more effective than in phosphate-buffered saline (PBS) buffer, as demonstrated by the degree of lysine glycation. Older studies have shown that glycated β2-microglobulin, BSA, myelin, and LDL induce the chemotaxis and secretion of TNF-α, IL-6, and IL-1β in human-derived monocytes [161,162,163]. Exposure of human monocytes to MGO caused led to increased migration of the cells [158], ROS levels, and decreased adhesion of monocytes to collagen. All the observed effects were prevented by curcumin [164].

On the contrary, the study of Valencia et al. showed that AGES are not inducers of proinflammatory response [165]. More particularly, Valencia et al. prepared glucose-, fructose-, ribose-, glyoxylic acid-, and GA-modified BSA. The researchers investigated the correlation between the binding of AGE preparations to RAGE with their ability to cause an inflammatory response in human peripheral blood mononuclear cells (PBMCs). In parallel, the researchers characterised the AGE preparations by different biochemical assays.

Murine monocytes from mice treated with modified BSA showed an increased proportion of proinflammatory monocyte phenotype accompanied by an increased gene expression of proinflammatory cytokines such as IL-6 and TNF-α. However, no change was observed in the expression of anti-inflammatory cytokines [166]. However, the exact modifications that BSA underwent were not clear in this study. In the same study, they observed increased proliferation and expression of TNF-α, IL-6, which was MAPK dependent as proven by the incubation with MAPK inhibitors [166]. In another study, exposure of mouse peritoneal macrophages to glucose-, MGO-and glyoxylic acid-modified BSA increased the TNF-a secretion. Among the three types of modified BSA, MGO-modified BSA was the most potent, as shown by the highest response [167].

#### 3.1.3. Neuronal Cell Lines

The loss of neuronal function and neuronal tissue is characteristic of any ND. Neurons can be indirectly affected by AGEs via the induction of inflammation by microglia, or they can be directly affected by AGEs. To that extent, the direct effects of glucose- and glyceraldehyde-derived AGEs were also described in in vitro studies with animal and human neuronal cells [168,169,170,171,172]. The following section will discuss the most common animal and human immortalised cell lines and primary cells used.

- Animal neuronal cell lines

The PC12 cell line is a pheochromocytoma-derived cell line from the rat adrenal medulla. This line is easy to handle and homogenous [173], while the cells can undergo neuronal differentiation in response to nerve growth factors resulting in large numbers of post-mitotic cells [174]. Many researchers have worked with this line to study neurodegeneration [175,176] and neurotoxicity caused by AGEs [177,178,179,180]. PC12 cells express RAGE [179] and they were vulnerable to ribosylated-BSA induced cytotoxicity, while the exposure increased iNOS and COX-2 expression and phosphorylation of p38 [178]. Cell apoptosis and expression of RAGE and NF-κB were significantly increased in PC-12 cells exposed to glycated BSA [179]. In another study, researchers showed that exposure of the cells to a mixture of glucose and MGO resulted in increased oxidative stress as reflected by the increasing levels of protein carbonyls, ROS, RAGE receptor, and NF-κB protein expression [177].

The mouse neuroblastoma cell line, Neuro-2a (also found in the literature as N2a), is a cell line widely used in neuronal differentiation studies and AGE-cytotoxicity studies [99,181,182,183,184,185]. Differentiation of Neuro-2a cells is achieved with retinoic acid, and the process is characterised by the outgrowth of neurites (axon-dendrite form) accompanied by a decrease in proliferation. In addition, this type of cell expresses RAGE [186]. In a study where Neuro-2A cells were exposed to a conditioned medium from microglial cells incubated with glycated BSA, significant cell death of Neuro-2a cells was observed. In addition to that, the neuronal cell viability was improved in the presence of aminoguanidine, a molecule that prevents the formation of AGEs [99]. Another study showed that the AGE-precursor MGO caused increased production of ROS and increased accumulation of intracellular CML (determined by anti-CML antibody) and apoptosis [181,182].

- Human neuronal cell lines

The SH-SY5Y cell line is a fast-growing neuroblastoma cell line derived by a bone marrow biopsy of a neuroblastoma patient with a sympathetic adrenergic ganglial origin [187]. This line has been extensively used to study mechanisms of neurodegeneration and especially PD as it was shown that this cell line can reproduce the dopaminergic phenotype of the disease and accommodate the formation of Lewy Bodies (LB)-like structures when exposed to α-synuclein [188,189,190]. Due to its characteristics, this microglial cell line is used to study the possible effects of AGEs [169,170,171,191,192,193,194,195,196,197,198,199]. SH-SY5Y cells express RAGE and in response to exposure to commercially prepared AGE-BSA they produce ROS and undergo apoptosis, via NF-κΒ and AMP-activated protein kinase (AMPK) activation [195]. In another study in which researchers incubated SH-SY5Y cells with MGO, the first-line defense antioxidant enzymes: superoxide dismutase (SOD), catalase (CAT), and glutathione peroxidase (GPx) were decreased while increases in MDA were detected. Mitochondrial damage and cytotoxicity were additional effects caused by MGO [170]. In addition, SH-SY5Y cells exposed to ribosylated α-synuclein showed increased levels of ROS [200].

Although SH-SY5Y cells are widely used in neurodegeneration research related to AGEs, some researchers point out the lack of a standardised protocol for cultivating these cells, which may result in inconsistent experimental outcomes [188,201]. The protocol followed by each researcher can lead to the loss or gain of different neuronal phenotypes such as dopamine-, noradrenaline-, acetylcholine-, glutamate-, serotonin-, or histamine-like phenotypes [202]. Furthermore, their differentiation requires the presence of Extracellular Matrix (ECM) proteins, neurotrophic factors, and serum, and different concentrations or sources of serum can have different results [203,204]. Furthermore, it appeared challenging to differentiate the SH-SY5Y cells into postmitotic neurons, while due to its cancerous origin, the line seemed to have an unstable genome [205]. SH-SY5Y cells express RAGE receptor [206] and interestingly the intracellular formation of AGEs (measured by immunoblotting) and apoptosis were shown in SH-SY5Y cells treated with glyceraldehyde (GA) [100,171]. However, the detection of AGEs was based on an immunoassay, and the researchers claim that the antibody used recognised unique GA-AGEs [100]. In the research work of Nasu et al., the identification of intracellular AGEs was performed not only by immunoblotting such as in the majority of these studies but was confirmed by mass spectrometry analysis [171]. In addition, the researchers observed, an increase in phosphorylation of tau protein and other AD markers such as VEGF and TGF-β. Moreover, a decrease in GAPDH activity was observed. Previously, it was shown that GAPDH activity is decreased in AD patients, and the reduction was associated with apoptosis [172,207,208].

Apart from the SH-SY5Y line, the Lund human mesencephalic (LUHMES) cells originated from a healthy 8-week-old embryonic mesencephalic tissue. The cells were immortalised by inserting the v-myc-vector under the control of a tetracycline-responsive promoter. LUHMES can be differentiated to post-mitotic dopaminergic neurons upon incubation with neurotrophic factors. However, due to the relatively low yield of differentiated cells, many researchers investigate different protocols to improve the differentiation efficiency [209]. This cell line has been increasingly used in neurotoxicology and PD studies [209,210]. LUHMES cells express RAGE [122] and when exposed to commercially prepared glycated-BSA showed increased levels of RAGE, phospho-p38, pERK1/2, pSAPK/JNK, and proapoptotic Bax protein. Thus, researchers concluded that glycated BSA promoted a RAGE-pp38-, pERK1/2-, and pSAPK/JNK-Bax-mediated apoptosis [122].

- Primary neuronal cells

Human primary cultures of neuronal cells are challenging to obtain since the material can only be obtained from postmortem brain tissue. Moreover, the procedure to isolate those cells from the brain is complex, and the yield of isolated cells is often low, making their use in neurodegenerative research limited [211]. A study with human primary neuronal cultures demonstrated that commercially glycated BSA induced cell apoptosis via RAGE-MAPKs-Bax pathways [97]. Primary rat cortical neurons treated with different kinds of AGE-modified BSA (glucose, D-glyceraldehyde, glycolaldehyde, MGO, GO) showed increased cell death with the highest cell death observed with D-glyceraldehyde modified BSA, which was also accompanied by a decrease in GAPDH activity. A specific antibody against D-glyceraldehyde modified BSA prevented neuronal death and the inactivation of GAPDH. The researchers also showed that the D-glyceraldehyde-modified BSA was the most neurotoxic among the different AGE molecules [172,212].

#### 3.1.4. Brain Endothelial Cells

Disturbances of the BBB are linked to several neurological diseases. Therefore in recent years, there has been an increasing amount of studies focusing on understanding the mechanisms that rule the integrity of the BBB. In vivo BBB studies are not easy. The complex network of the BBB makes it challenging to isolate and study the underlying mechanisms but also to develop suitable in vitro models. The BBB comprises a physiological entity that consists of a basal membrane, the brain microvascular endothelial cells (BMECs), pericytes, astrocytes, and microglia. However, brain endothelial cells play a crucial role in maintaining the brain barrier function through the junctional complex consisting of tight junctions proteins and adherens junction proteins [213]. Therefore, to mimic the BBB with in vitro studies, primarily endothelial cells are used. In the following section, the most commonly used brain endothelial cell lines will be presented.

- Animal-derived immortalised cell lines

The RBE4 line is a rat cerebral brain endothelial cell line immortalised with the adenovirus E14 gene, which was used as an in vitro model of BBB [214,215,216]. This cell line expresses adherens junction proteins such as E-cadherin, adhesion molecules such as ICAM-1, tight junction proteins such as occludin, and the specific BBB enzymes γ-glutamyl transpeptidase and alkaline phosphatase (ALP) [214]. Despite the fact that this line expresses RAGE [217], it has been rarely used in studies with AGEs.

A more commonly used brain endothelial cell line is the mouse bEnd.3 line. This line is derived from mouse primary endothelial cells immortalised with the Polyoma T antigen [218]. Comparative studies have shown that this cell line exhibits lower transepithelial electrical resistance (TEER) values than primary models [219,220]. Moreover, this cell line expresses RAGE [221] and has been extensively used as a model of the BBB to study the effect of neuroprotective molecules and the effect of Aβ peptide on the BBB [222,223,224,225,226,227]. However, also for this cell line, studies on AGEs and their impact on the BBB are limited. MGO-modified BSA increased the permeability of the endothelial barrier of bEnd.3 cells in vitro and induced oxidative stress by impairing the mitochondrial function [59].

- Human derived immortalised cell lines

The human hCMEC/D3 cell line is derived from primary microvessel endothelial cells immortalised with SV40 and hTERT lentiviral transduction [228]. Due to its human origin, this cell line is widely used to study transporters and receptors for drug delivery to the brain [229,230,231,232]. An extensive characterisation of this cell line showed that the cells express endothelial and BBB markers and have a highly restricted permeability to molecules, which is closer to the physiological frame than animal immortalised brain endothelial cells [228]. In the context of AGE research, this line has been hardly applied as a model of neurodegeneration. Nevertheless, it was shown that RAGE is expressed in these cells [221].

- Primary endothelial cells

Brain Microvascular Endothelial Cells (BMECs) are primary cells with unique properties that include the expression of tight junction proteins and polarised expression of transporters, receptors, and enzymes [233,234]. However, some main challenges arise from the use of primary BMECs: the difficulty in isolating sufficient cells, the risk of contamination by other cells, the use of expensive growth factors to maintain them, and the interindividual variability. Brain vasculature accounts for only 0.1% (*v*/*v*) of the brain tissue, and therefore to obtain sufficient amounts of BMECs, many rodents have to be sacrificed [235]. Cells from larger species are used to overcome this problem, such as cows, pigs, or non-human primates. Human primary BMECs are needed to have a physiologically relevant model for humans. However, the acquisition of such cells is difficult for ethical reasons, and therefore, their use is scarce. Only a few studies investigated AGEs using BEMCs. One of them studied the effects of commercially prepared AGE-BSA on the production of tight junction proteins such as zonula-1 (ZO-1) occludins, and claudins. Protein expression of claudin-5 decreased whereas occludin and ZO-1 remained unchanged. In addition AGE-BSA caused significantly decreased TEER values and increased permeability. Ocludin-5 levels increased when BMECs were treated with RAGE siRNA. The study also showed that AGEs decrease the cellular levels of occludin by increasing the expression of VEGF and matrix metaloproteinase-2 MMP-2 (profibrotic factors, they induce basement membrane hypertrophy resulting in thickening and ultimately disruption of the barrier) [58].

The Human Umbilical Vein Endothelial Cells (HUVECs) are primary endothelial cells isolated from human umbilical cords. Due to their origin, these cells are more easily accessible types of endothelial cells than other types of blood vessel endothelial cells. Standard protocols for the isolation and maintenance of these cells exist, or they can be obtained from commercial resources. HUVECs express RAGE [236] and endothelial markers such as ICAM-1, VCAM-1, tight junction proteins such as ZO-1, and connexin proteins [237,238,239]. Due to their availability, HUVECs have been used as an in vitro BBB model to study the effects of several compounds, including AGEs, on BBB integrity. Glycated insulin significantly decreased the viability of HUVECs, by increasing the expression of Bax while decreasing the expression of Bcl-2 and increasing the production of ROS and permeability of the monolayer [57]. In another study, HUVECs exposed to food-derived AGE extracts, glycated BSA, MGO modified BSA, MGO modified ovalbumin, or CML-modified BSA decreased the activity of GSH and increased the activity of glutathione peroxidase (GPx). In both cases, MGO modified BSA induced the most significant decrease in GSH activity and increase in GPx activity compared to the other glycated proteins, whereas, in the presence of N-acetyl-cysteine (NAC) and aminoguanidine, the effects were reversed [167]. In another study, MGO exposed HUVECs showed increased ROS production and apoptosis accompanied by an increase in Bax protein expression and a decrease in Bcl-2, increased phosphorylation of the MAPKS (p38, JNK, ERK), and decreased NF-κΒ activation [240,241]. In addition, Glyoxalase-1 (GLO-1) protein levels decreased while Nrf-2 remained unchanged (isosamidin counteracted all the effects) [241]. HUVECs incubated with MGO showed a significant increase in ROS production, monolayer permeability, actin stress fibers, and a decrease in gene and protein expression of ZO-1, Cx43, and the gap junctional intercellular communication (GJIC) [237].

To sum up, monocultures are an extremely useful tool for understanding fundamental cellular effects and studying cytotoxicity. Despite their simplicity, they provide a good starting point to investigate MoA concepts. An overview of the findings regarding the exposure of the abovementioned cell types to AGEs or other RAGE ligands is summarised in Table 1.

### 3.2. Reporter Cell Lines

Reporter cell lines are very useful in vitro tools for MoA studies as they allow tracking of the expression of certain genes upon exposure to specific stimuli. Hence, they can provide important information about the activation of cellular pathways. Many reporter gene cell lines are currently available, and several were also applied in studies to investigate the activation of relevant pathways by AGEs.

A rat C6 glioma reporter cell line was developed after transfection of the cells with plasmid vectors encoding luciferase under the control of the enhancer of the NF-κB gene and human full-length RAGE cDNA. Incubation of these reporter cells with HMGB1 induced the NF-κΒ luciferase activity [244,245]. Furthermore, glucose and glyoxylic acid-modified BSA significantly increased the activation of NF-κΒ luciferase activity in osteoblasts and vascular smooth muscle cells, compared to the control BSA (no RAGE inhibitors were used to prove that this was due to AGEs) [246,247]. Considering the AGE-induced ROS production in cells, specific reporter gene assays could give more insight into the cytotoxic effects of glycated proteins and the potential protective effect of several compounds against AGE-toxicity. Once cells are exposed to electrophilic reactive molecules, the nuclear transcriptional Nrf2 gets activated and binds to the electrophile responsive element (EpRE). The EpRE, also known as antioxidant response element (ARE), is involved in the gene expression of antioxidant enzymes such as glutathione S-transferase (GST), HO-1, and NAD(P)H quinone oxidoreductase 1 (NQO-1) [248,249,250,251]. The EpRE-LUX reporter cells are Hepa-1c1c7 mouse hepatoma cells transfected with a plasmid containing the luciferase gene under the control of the EpRE-enhancer element in conjunction with a minimal promoter and an initiator [252]. In addition, the Nrf2 HepG2 reporter cell line was also developed [249]. Another line is the U-2 OS Nrf2 CALUX human osteosarcoma cell line transfected with a vector containing four different EPRE sequences: under the promoter-less luciferase reporter-construct pLuc [253].

Moreover, the NF-κΒ-RE-luc2P consists of HEK293 cells transfected with a vector containing a luciferase gene under the control of a minimal TATA promoter with multiple Nuclear Factor-κB-response elements (NF-κB-REs) [254]. In a study, THP-1 cells were transfected with a pNF-κB Luc plasmid. The NF-κB activation upon exposure to S100b and the inhibition of this activation by LR-90 (an AGE inhibitor) was shown [154]. A THP-1 reporter cell line was developed by transfecting THP-1 cells with a recombinant plasmid containing luciferase gene under the control of the promoter of the COX-2 gene. Results showed the involvement of NF-κΒ in the transcription of COX-2 in the presence of S100b and AGEs [159].

Overall, reporter cell lines can be of great use to study the toxicity and the activation of specific transcription pathways by AGEs. However, the lack of brain-specific reporter cell lines is a clear limitation for using such models in AGE-mediated ND research. Therefore there is an urgent need to develop brain-related reporter lines and even an AGE-RAGE reporter cell line.

### 3.3. iPSCs

In 2006, Yamanaka’s research group announced the successful reprogramming of mouse fibroblasts into induced Pluripotent Stem Cells (iPSCs) by transducing the fibroblasts with selected genes. Due to their pluripotency, iPSCs are self-renewable, and they can differentiate into different cell types. The discovery of iPSCs offered a unique possibility to develop in vitro human disease models as iPSCs allowed researchers to generate multiple human differentiated cell types with the same genotype. At the same time, iPSCs, help researchers to overcome challenges related to the availability of human material. iPSCs can be differentiated into neurodegeneration-relevant cell types, and up to now, several studies have demonstrated the effectiveness of using iPSCs as an in vitro model for neurodegenerative diseases such as PD [255,256,257,258], AD [259,260], and ALS [261,262]. The efforts to develop models of neurodegenerative diseases are based on the generation of iPSC-derived microglia [263,264], neurons [258,265], and cell types composing the BBB, such as brain endothelial cells [266,267], astrocytes [268], and lately pericytes [269,270,271]. A considerable effort has also been made towards BBB modelling with iPSCs. iPSCs derived BMECs exhibit in vivo-like barrier phenotypic characteristics such as the expression of BMEC-specific proteins, polarised cells, high barrier integrity (TEER), and low barrier permeability [272,273]. Haenseler et al. assessed the resemblance of iPSC-derived microglia co-cultured with cortical neurons with human fetal microglia by conducting transcriptome analysis [274]. RNA-seq data combined with microarray data showed that iPSC-derived microglia expressed key microglia and neurodegeneration-related specific genes. The research work of Υagi et al., 2011 firstly demonstrated the application of patient-derived iPSCs with mutations in disease-relevant genes such as presenilin-1 (A264E) and presenilin-2 (N141I). Cells appeared to have excess deposition/secretion of Aβ [275]. Later on, researchers developed iPSC-derived neurons from nerve cells of AD patients with mutations in the presenilin gene [260]. In addition, iPSC-derived neurons from AD patients exposed to conditioned media produced by activated primary murine microglial cells showed a reduction in cell viability and neurite length due to increased oxidative and inflammatory responses [276]. These results stress the importance of cell communication and cross-talk in health and disease, which could be investigated using co-culture presented in the following section.

Overall, despite the strength of iPSCs models (derived from human material), differentiated cell lines from iPSCs are time-consuming, non-high-throughput, and expensive models. Their application in the context of neuroinflammation and neurodegeneration induced by AGES is an unexplored research area that may provide researchers with valuable human in vitro models.

### 3.4. Two-Dimensional Co-Cultures

Cell-to-cell communication is vital in the maintenance of normal physiology. Thus, research in neuroinflammation and neurodegeneration evolves around the interaction and cross-talk between different cell types (neurons, microglia, endothelial cells). For instance, a study showed that conditioned medium from pericytes exposed to AGE-BSA and pre-incubated with an anti-TGF-β1 antibody recovered the TEER and protein expression of claudin-5 of BMECs, suggesting the involvement of TGF-β1 (secreted by microglia) in the disruption of BBB by AGE-BSA. Hence, developing in vitro model systems to understand the mechanisms involved in this cross-talk is a significant area of research. Several studies over the past years have attempted to model the neuro-immune axis communication and the complex in vivo situation by setting up in vitro co-culture systems. While in vivo studies better mimic the physiology of the CNS, one of the benefits of co-culture in vitro systems lies in the fact that they are a more simplified representation of the physiology, which can help to understand better the underlying molecular mechanisms and enable researchers to answer more complex research questions. In addition, researchers can experimentally manipulate each cell type and elucidate the contribution of each cell type in the interactive mechanisms. However, determining the optimal conditions under which each distinct cell population can co-exist and maintain its normal phenotype is crucial for establishing a good in vitro co-culture model [277].

Co-culture models are a standard tool used in studies on the neuro-immune axis and BBB. In the neuro-immune axis research, neurons and immune cells are the most common cell types used in co-cultures. In this context, studies have shown the bidirectional communication between microglia and neurons. Many studies describe the role of activated microglia in neuroinflammation and consequently neurodegeneration [278,279]. Microglia affect neurons by secreting pro-inflammatory and oxidative factors, while neurons respond by secreting neuropeptides [280]. Therefore, many studies have developed co-culture model systems with neurons and microglia, either in a mixed culture or separated by a porous membrane (transwells) [107,281,282,283,284]. Transwells are an extensively used system in co-culture cell models. It consists of a luminal and a basal compartment separated by a microporous membrane that allows the transfer of molecules from one compartment to the other. Therefore it provides compartmentalisation of cells while allowing paracrine communication between the distinct cell types in the system. For instance, the co-cultures in transwells of the following cell type combinations were described in the literature: BV2 and SH-SY5Y [285], BV2 and Neuro-2A [286], BV2 and THP-1 [287], HMC3 and SH-SY5Y [288], primary neurons and microglia [289], triculture of N11 and Neuro-2A and brain endothelial cells [290], triculture with primary neuron and astrocyte and microglia [291]. The MGO-derived MG-H1 was found to increase the adhesion of THP-1 monocytes to human umbilical vein endothelial cells (HUVECs) [243].

Similarly, in BBB modelling, the co-culture of brain endothelial cells with other cell types of the BBB such as neurons, microglia, and/or pericytes were described. Interestingly co-culture of brain endothelial cells with different cell types of the BBB was shown to improve the integrity of the endothelial barrier [290]. For example, the presence of astrocytes and pericytes [273], iPSCs derived astrocytes and neurons, and neuronal stem cells were found to improve the barrier phenotype of BMECs in vitro [273,292,293]. In addition, the following co-cultures of BBB models were also described: bEnd.3 and primary astrocyte cultures [226], and rat primary astrocytes cultured with rat brain endothelial RBE.4 cells [294]. Especially in the field of glycation and neurodegeneration, it would be of great interest to study the effects and the translocation of glycated molecules across the BBB and by using in vitro co-culture models. For example, in a study with glycated insulin, the viability and apoptosis of HUVECs in co-culture with astrocytes in a transwell system were investigated [57]. Insulin modified with glucose reduced the viability of both cell types, increased ROS levels, pro-apoptotic proteins (Bax), and permeability to HUVECs. The researchers claim that the observed apoptosis could be attributed to a weakened BBB due to glycated insulin and therefore increased permeability and disturbance of barrier homeostasis. However, to the best of our knowledge, the application of co-cultures in brain-related studies on AGEs is currently limited.

Overall, the models described in this review’s previous sections are described with the general term two-dimensional (2D) cell culture systems since cells grow on a flat surface. Models based on 2D cultures were proven beneficial to understand MoA concepts in neurodegeneration. The 2D cultures are well-established models, relatively easy to understand and analyse, fast, and not expensive. All these characteristics make them popular in cell-based in vitro studies.

However, the limitations of such models relate to their simplicity which does not recapitulate the complex architecture of the brain cellular environment in total. More precisely, the lack of multiple cell type interaction and an organised neuronal network are important drawbacks. Moreover, the pathological phenotypes of ND, such as the presence of neurofibrillary tangles and Aβ plaques, are an additional limitation [295].

### 3.5. Three-Dimensional Models and Microfluidics

ND share common features characterised by multiple complications involving protein misfolding, aggregation, inflammation, toxicity, aging, and genetic mutations. Thus many different cell types and pathways are involved. Therefore, the development of multiple representative in vitro models is essential to study the multidimensional causality of ND. Late-stage hallmarks of ND such as amyloids plaques and tau protein tangles cannot yet be recapitulated by simple cell lines or by patient-derived iPSCs [296].

In the effort to develop in vitro models with increasing complexity that mimic the architectural, spatial, and cellular complexity of the human brain, three-dimensional (3D) models of cerebral organoids have drawn the attention of researchers in the last decade. Cerebral organoids are generated from iPSC from individuals with a genotype or phenotype of interest. Then, iPSCs are differentiated towards the desired cell type, i.e., neurons, microglia, and astrocytes [297]. Cerebral organoids are self-organising structures grown in a gelatinous laminin-rich extracellular matrix called matrigel. The development of brain organoids helps capture the cellular diversity and recapitulate the spatial arrangement of the brain. Different protocols and culture conditions drive organoids to differentiate into discrete brain tissue-specific areas [298]. Cerebral organoids are a very useful tool to recapitulate the formation of extracellular proteins, as observed in ND. Due to the presence of matrigel 3D brain organoids offer an environment in which the aggregation of proteins is facilitated. For instance, Kim et al. generated midbrain PD organoids and observed the accumulation of α-synuclein [299]. Another 3D organoid AD model showed increased extracellular deposition of Aβ aggregates and hyperphosphorylated tau protein [300].

Brain organoids are an undoubtedly significant development in 3D cell culture models with brain-like structural characteristics and patient-based genetic background. Despite their application and potential in neurodegeneration research, they present some limitations. More precisely, brain organoids lack vasculature and immune cells, they also present heterogeneity, and neurons show a relative immaturity that resembles the early developmental stages of the human brain (2nd trimester of the fetal brain) [298]. Thus, brain organoids might have less application to late-onset neurodegenerative diseases. Given that the immune and the vascular system are essential factors to consider when studying the role of AGEs in neurodegeneration, the validity of brain organoid models might be limited.

Efforts towards the vascularisation of the brain organoids have been made [301,302]. This kind of vascularised organoid system could provide opportunities to model the BBB in 3D. Researchers have also tried to tackle the lack of immune cells in brain organoids by incorporating microglia into an organoid culture [303]. However, such models are difficult to establish and interpret compared to the conventional 2D co-cultures. Another limiting factor of brain organoids is the presence of LPS in matrigel, which can be a confounding factor when studying immune responses by AGEs.

Most in vitro neurodegeneration models used in current research are based on 2D and 3D cell cultures. However, the drawbacks of 2D and 3D models involve the lack of mechanical and biochemical microenvironments that build the complex structure of human organs, including the brain. Microfluidic systems (also known by the term organ-on-a-chip systems) can overcome these limitations of conventional cell culture models. Especially for BBB models, microfluidic systems in which microchannels connect the two cellular compartments have been developed, such as neurons and glial cells [304,305]. These advances are especially relevant for BBB models. Many advances have been made in microfluidics technology to constitute a 3D model of the BBB by using human brain endothelial cells or a combination of endothelial cells with neurons and microglia [306,307]. Commercially available BBB-on-a-chip provides the opportunity to customise the system based on the research question [306,307]. For example, the co-culture of microglia with brain endothelial cells in such microfluidic systems is possible.

Microfluidic settings cover parameters such as fluidic stress, paracrine and juxtracrine signalling, characterisation of cell types, and ECM scaffold (tissue-specific) composition [308]. In addition, microfluidics is considered less expensive than conventional cell culturing as they require fewer cells and less medium. This aspect is essential when using iPSC-derived cells as the procedure is lengthy, expensive, and often not highly efficient [308]. Microfluidic systems of neurons and microglia were reported [304].

As explained earlier, iPSC-derived cells’ use is a promising direction towards a better understanding of ND. Applying these cells into the field of microfluidics offers the unique opportunity to use patient-derived material in in vivo-like physiology models. Studying the role of AGEs in neurodegeneration and especially on the BBB function with BBB-on-a-chip system could be an interesting area to explore.

## 4. Advisable In Vitro Models for AGE-Related Research

Table 2 gives an overview of the advisable in vitro models grouped by their relevance to the specific endpoint. More precisely, as a model of neuroinflammation, the human microglial line HMC3 stands out as it presents all the macrophage-like characteristics needed to study potentially inflammatory responses from AGEs. In addition, the HMC3 cell line express RAGE and was used as a model of neuroinflammation in many AGE-related studies. In AGE-related neurotoxicity studies, the neuronal cell line SH-SY5Y presents the characteristics that resemble the pathophysiology of neurodegeneration. Additionally, this neuronal line is widely applied in the field of AGEs, and it was shown that this neuronal line can accumulate AGEs intracellularly. Regarding the cellular models of BBB, the human brain endothelial cell line hCMEC/D3 is currently the best characterised brain endothelial model, which exhibits all the junctional endothelial properties and RAGE expression.

As discussed above, co-cultures in a transwell system provide a more physiologically relevant model. Firstly, because the transwell system allows the study of paracrine interactions between cells and secondly because the spatial organisation of the system mimics a physical barrier. The application of co-cultures in transwells of neuronal lines with microglial lines is a commonly used model of neuroinflammation in the literature. A suitable model to study AGEs in neuroinflammation is the transwell co-culture system with the HMC3 microglia and the SH-SY5Y neuronal line, presented in a recent study [309]. Especially for the BBB, which by definition describes a physical and functional barrier consisting of multiple types of cells, the co-cultures of brain endothelial cells with other relevant cells such as pericytes in a transwell system could be a good in vitro tool. However, currently, fundamental questions regarding the impact of AGEs on the integrity of BBB and the kinetics of AGEs across the BBB remain elusive. Therefore, simpler models could give a better understanding of the basics of AGEs translocation and BBB compromise. In addition, concerning the abovementioned questions that remain unclear about AGEs and BBB disruption, the BBB on a chip is a great tool to study these effects.

**Table 2:** The advisable in vitro models per endpoint of interest. The endpoints in which the role of AGEs in neurodegeneration can be studied are neuroinflammation, neurotoxicity, and BBB.

Advisable Model per Endpoint		Advantages	Disadvantages	References
Neuroinflammation	HMC3 microglial line	Human line, RAGE expression, M1 and M2 phenotype, existing literature on AGEs effects	No aggregate formation	[128,129,132,133,310]
	Co-culture of neurons with microglia in transwell	Well-established models, they give insights into how AGE indirectly affects neuron viability by activating microglia, easy to establish and interpret, comparative studies on the responses, i.e., SH-SY5Y/HMC3	No aggregate formation	[285,286,287,288,290,309]
	iPSC-derived microglia	Patient-derived cells with diseased genotypic background, formation of aggregates	Non-high throughput, expensive, laborious procedure, low efficiency of differentiated cells	[263,264]
	NF-κΒ reporter cell lines	Very informative to understand AGE signalling	Most of the available lines are no brain-related lines	[154,159,244,246,247,254]
Neurotoxicity	SH-SY5Y	Human line, extensively used in AGE-studies, RAGE expression, intracellular formation of AGEs, differentiated to dopaminergic neurons, Lewy body formation observed	Multiple differentiation protocols exist, possible unstable genome due to cancerous origin	[100,170,171,188,189,192,195,197,200,205]
	iPSC derived neurons	Patient-derived cells with diseased genotypic background, formation of aggregates	Non-high throughput, expensive, laborious, low efficiency	[258,265]
	Nrf-2 reporter cell lines	Very informative to understand AGE signalling and the potential protective effects of AGE inhibitors	No brain-related lines are available	[249,252]
BBB	hCMEC/D3	Human line, expression of endothelial junctional markers and transporters, RAGE expression, applied in AGE and ND research, widely used and characterised BBB model	Exhibit lower TEER than primary endothelial cells	[311]
	iPSC derived brain endothelial cells	Patient-derived cells with diseased genotypic background, formation of aggregates, high TEER values	Non-high-throughput, expensive, laborious, low efficiency, reproducibility not confirmed	[267,272]
	Co-cultures of brain endothelial cells with microglia in transwells	Well-established models, paracrine communication between cell types, more representative of BBB physiology, easy to establish and interpret	No aggregate formation, complicated	[57,226,273,292,293,294]
	BBB-on-a-chip	3D, fluid shear stress, ECM, paracrine and juxtacrine signalling, combination of cell types	Complex	[304,305]

Reporter cell lines elucidate the activation of certain signalling pathways after a specific stimulus. Even though currently there are no available brain-related cell lines, the existing reporter cell lines can provide us information regarding the initiation of pro-inflammatory or anti-inflammatory pathways in the cells after their exposure to AGEs.

The application of iPSC-derived cells allows working with human-derived material, which eventually carries the genotypic background of diseased individuals. For that reason, the application of iPSCs in ND research is growing in the literature; different protocols for iPSC-derived microglia, neurons, and brain endothelial cells were described. Thus, it would also be an interesting tool to apply in AGE-related neurodegeneration research.

## 5. Conclusions and Main Challenges in AGE In Vitro Research

The main goal of this review was to present and discuss in vitro models that were applied or have the potential to be used in research on AGEs and ND. We first introduced and explained the current knowledge on AGEs regarding their formation and accumulation in the human body. Then, we presented the existing evidence linking the involvement of AGEs in the ND and explained the basic concepts of brain physiology and immunology affected by AGEs. Next, we presented and discussed the available in vitro models to study AGE-mediated neurodegeneration by dividing them into sections from simple models, which have been applied to more complex models that have not been yet applied in the field of AGEs but they could offer opportunities in the future. Finally, we gathered all the advisable in vitro tools based on their relevance to the three primary endpoints that AGEs can impact the brain pathophysiology and their characteristics and suitability to mimic ND pathophysiology.

MR and MRPs/AGEs are a contemporary yet controversial research topic between the food industry and food and health research. Therefore, a large volume of studies aim to investigate the effects of AGEs on human health. Despite the large volume of in vitro studies focusing on understanding the role, formation, and kinetics of AGEs, variable factors during the preparations of AGEs make the comparison between studies complex and could create discrepancies in the observed effects. For example, different protocols of preparation include various types of buffers (PBS or other phosphate buffers), different types of glycation agents (glucose, fructose, ribose, MGO, GO), time of incubation, temperature, and way of heating (wet or dry glycation). In some studies, even commercially prepared AGEs were used instead of “lab-made” AGE preparation under known and controlled conditions.

Another point often neglected in the literature is the good chemical and structural characterisation of AGE preparations. Most of the existing studies use immuno-based assays to characterise the product instead of analytical techniques. Furthermore, concerning the need for better characterisation techniques of AGE preparations, this issue becomes relevant for characterisation and understanding fundamental questions that remain answered regarding the origin of AGEs in the brain. Several studies have indicated the intracellular formation of AGEs by microglia or neurons, but the identification of intracellular AGEs in those cases is made by immuno-assays, which have received much criticism regarding their reliability to identify and quantify AGEs [94,96,98,100,171]. The concerns about these techniques are mostly related to the undefined specificity and affinity of the anti-AGE antibodies [312,313]. Therefore, such results must be interpreted with caution. Despite that, researchers often prefer immune-based assays with anti-AGE antibodies as it is a fast, easy to perform and interpret method. To better and more precisely characterise AGEs, LC/MS protocols have been developed as the most reliable method to identify AGEs in different matrices [17,151,313,314,315,316,317]. Metabolomics/proteomics approaches using LC/MS technology are considered the most robust and insightful tool to “fingerprint” AGE preparations and answer questions regarding the intracellular formation of AGEs. For this reason, LC/MS technology was described as the “gold-standard” methodology for AGEs characterisation. Related to the sample characterisation and equally important is the binding of AGE preparations to the RAGE receptor. In most studies, the observed effects are often attributed to RAGE activation, but the binding to the receptor is not proven and is instead assumed.

Another critical point is the presence of LPS in AGEs preparations. LPS is a burden in studies where the immune responses are investigated. Most of the existing studies show that AGEs can induce immune responses in monocytes/macrophages or microglia. However, several other studies found contradictory results. A study demonstrated that glycated HSA did not stimulate cytokine secretion in PBMCs [318]. In this particular study, researchers point out that only in the presence of a low concentration of endotoxins, AGEs were capable of causing a release of cytokines to PMBCs. A study on human lung cells showed that glycolaldehyde modified β-lactoglobulin was unable to stimulate the gene expression of inflammatory cytokines after treating the preparations with Triton X-114 to remove LPS [319]. Another study has shown that CML modified beta-lactoglobulin and HSA could not bind to RAGE receptor and could not also induce the gene expression of IL-6, IL-8, and TNFα in lung epithelial cells, coming in agreement with other studies suggesting that CML modified proteins are not RAGE ligands [165,320]. Of note is that in both studies, the researchers removed the LPS content of the glycated samples after the glycation procedure by using the Triton x-114 protocol [321]. However, it is not known to what extent glycation is affected by the treatment with Triton X-114. Thus, few studies have shown that removing LPS from the preparations removes the pro-inflammatory properties of AGE preparations [165,319]. Such studies stress the importance of LPS content control when studying immune responses from AGEs.

Clinical studies point to the association between the accumulation of AGEs in the brain of ND patients with the progression of the disease. However, whether AGEs are the cause remains to be answered. In addition, the source of the observed AGE accumulation in the brain of those patients (dietary or endogenous) is not yet fully understood. Regarding the intake of AGEs via diet, an elegantly designed animal intervention study showed that dietary CML could cross the BBB and accumulate in the brain [26]. Theoretically, the brain will only come in contact with dietary AGEs that have been digested and absorbed in the intestine. For that reason, studies on AGE digestion and absorption (i.e., in vitro digestion models) are crucial to understanding the type of dietary AGEs that will circulate and cross the BBB to reach the brain.

On the other hand, endogenous AGEs can also be formed due to increased glucose levels derived from the high glycemic diet. For example, albumin is an abundant protein in the human body that can undergo glycation due to excess glucose or other glycating agents (i.e., MGO). In the brain, such modified protein can cause inflammatory responses and contribute to a vicious cycle of inflammation.

Moreover, the brain is an organ that primarily depends on glucose as its main source of energy. Therefore there is increased glycolysis in the brain. Highly reactive molecules such as α-hydroxy aldehydes (glyceraldehyde and glycolaldehyde) and dicarbonyl compounds (glyoxal, methylglyoxal, and 3-deoxyglucosone) are formed as a result of glycolysis. Thus, such molecules in the brain can contribute to locally produced AGEs extracellularly or intracellularly.

Another significant challenge in the research field is the link of in vitro studies to the clinical/in vivo state, especially for the endogenous AGEs, as most clinical studies mainly focus on the fate and metabolism of dietary AGEs. Concerning that, the translation of the exposure to dAGEs based on consumption of certain foods is difficult to translate to a concentration that cells are going to be exposed to. Overall, the complexity and multiple sources of protein glycation require the application of in vitro models to understand the potential contribution to neurodegeneration.

## Figures and Tables

**Table 1:** Main outcomes from existing studies on AGEs or other RAGE ligands, in which monocultures were used as models of neuroinflammation, neurodegeneration, and BBB.

Relevant Endpoint	Cell Type	Line Name	Origin	Exposed to	Outcome	Reference
Neuroinflammation/inflammation	Microglia	BV2	mouse	MGO-BSA	↑ •NO, ROS, NLRP3, NF-κΒ, TNF-α, IL-6, MAPKs, RAGE, COX-2	[89]
		N11	mouse	Glu-BSA	Microglial activation, ↑ •NO, IL-6, MCP-1, TNF-α	[119,120,242]
				Glu-CEA	↑ •NO, iNOS, TNF-α, IL_6	[114,118]
		HMC3	Human	Glu-HSA	↑ •NO, apoptosis Anti-RAGE prevented cell death	[133]
				Glu-BSA	Activated microglia (↑ Glu-5, CR3/43), ↑ RAGE, TNF-α Anti-RAGE decreased TNF-α	[132]
		HMO6	Human	Aβ peptide	↑ RAGE, synthesis of AGE-albumin	[96,98,122]
	Monocytes/macrophages	THP-1	Human	Glu-BSA	↑ VEGF, IL-6, TNF-α, iNOS, RAGE, promoted M1 phenotype	[92,151,160]
				MG-H1	↑ Adhesion to HUVECs	[243]
				S100b	↑ RAGE, MCP-1, IP-10, COX-2, NOX2, O_2_^•^, NF-κB activation. LR90 blocked the expression of pro-inflammatory cytokines, NF-κΒ and decreased the adhesion of THP-1 to endothelial cells.	[154]
				Hypoxia	↑ AGEs, MCP-1, RAGE, NF-κΒ, Μ1 phenotype	[153,155,156]
				MGO	↑ secretion of AGEs, glycation of cell surface proteins, unchanged ROS and RAGE, ↑ IL-1β, IL-8, TNF-α ↑ migration, Impaired chemotaxis	[157,158]
				MGO-BSA	↑ COX2, TNF-α, M-CSF	[152,159,160]
Neurodegeneration/neurotoxicity	Neuronal	PC12	Rat	Ribosylated-BSA	↑ apoptosis, iNOS, COX2, pp38	[177,178,179]
				Glu-BSA	↑ apoptosis, RAGE, NF-κΒ	
				Glu + MGO	↑ protein carbonyls, ROS, RAGE, NF-κΒ	
		Neuro2A	mouse	MGO	↑ ROS, apoptosis, intracellular CML	[181,182]
		SH-SY5Y	human	AGE-BSA	↑ RAGE, ROS, apoptosis, NF-κB, AMPK	[195]
				MGO	↑ apoptosis, ROS, MDA, mitochondrial damage ↓SOD, CAT, GSH	[170]
				Ribosylated α-synuclein	↑ ROS	[200]
				GA	↑ AGE accumulation intracellularly, apoptosis, VEGF, TGF-β, phosphorylated tau ↓GAPDH activity	[100,171]
		LUHMES	Human	AGE-BSA	↑ RAGE, MAPKs, Bax	[122]
BBB	Endothelial	bEnd.3	Mouse	MGO-BSA	↑ monolayer permeability, ROS ↓SOD2 activity, impaired respiratory metabolism	[59]
		BMECs	Human or animal	AGE-BSA	↓claudin-5, TEER, Unchanged occludin-1 and ZO-1, ↑ monolayer permeability due to ↑ VEGF, MMP-2 leading to thickening of the basement membrane and barrier disruption	[58]
		HUVECs	human	Glu-BSA MGO-BSA MGO-OvA, CML-BSA	↓GSH activity ↑ GPx activity	[167]
				Glycated insulin	↓viability, Bcl-2 ↑ Bax, ROS, permeability	[57]
				MGO	↑ ROS, apoptosis, Bax, MAPKs ↓Bcl-2, GLO-1, unchanged Nrf2 ↑ monolayer permeability, actin stress fibers ↓ZO-1, Cx43, GJIC	[237,240,241]

↑: increased, ↓: decreased.

## Data Availability

No data were generated in this work, only reviewing of existing data.
